# Global DNA Methylation patterns on marsupial and devil facial tumour chromosomes

**DOI:** 10.1186/s13039-015-0176-x

**Published:** 2015-10-01

**Authors:** Emory D. Ingles, Janine E. Deakin

**Affiliations:** Institute for Applied Ecology, University of Canberra, Canberra, ACT 2601 Australia

**Keywords:** Epigenetics, X chromosome inactivation, Cancer, Genome stability

## Abstract

**Background:**

Despite DNA methylation being one of the most widely studied epigenetic modifications in eukaryotes, only a few studies have examined the global methylation status of marsupial chromosomes. The emergence of devil facial tumour disease (DFTD), a clonally transmissible cancer spreading through the Tasmanian devil population, makes it a particularly pertinent time to determine the methylation status of marsupial and devil facial tumour chromosomes. DNA methylation perturbations are known to play a role in genome instability in human tumours. One of the interesting features of the devil facial tumour is its remarkable karyotypic stability over time as only four strains with minor karyotypic differences having been reported.

The cytogenetic monitoring of devil facial tumour (DFT) samples collected over an eight year period and detailed molecular cytogenetic analysis performed on the different DFT strains enables chromosome rearrangements to be correlated with methylation status as the tumour evolves.

**Results:**

We used immunofluorescent staining with an antibody to 5-methylcytosine on metaphase chromosomes prepared from fibroblast cells of three distantly related marsupials, including the Tasmanian devil, as well as DFTD chromosomes prepared from samples collected from different years and representing different karyotypic strains. Staining of chromosomes from male and female marsupial cell lines indicate species-specific differences in global methylation patterns but with the most intense staining regions corresponding to telomeric and/or centromeric regions of autosomes. In males, the X chromosome was hypermethylated as was one X in females. Similarly, telomeric regions on DFTD chromosomes and regions corresponding to material from one of the two X chromosomes were hypermethylated. No difference in global methylation in samples of the same strain taken in different years was observed.

**Conclusions:**

The methylation patterns on DFTD chromosomes suggests that the hypermethylated active X was shattered in the formation of the tumour chromosomes, with atypical areas of methylation on DFTD chromosomes corresponding to locations of X chromosome material from the shattered X. The incredibly stable broad methylation patterns observed between strains and over time may reflect the overall genomic stability of the devil facial tumour.

**Electronic supplementary material:**

The online version of this article (doi:10.1186/s13039-015-0176-x) contains supplementary material, which is available to authorized users.

## Background

DNA methylation, the addition of a methyl group to convert cytosine nucleotides to 5-methylcytosine, is an epigenetic modification predominantly associated with regulating transcription and transposable elements (reviewed in [1]). Although DNA methylation is used as a regulatory mechanism in a broad range of eukaryotes, the extent of methylation and its location within the genome differs markedly between species. Despite being the most studied epigenetic mark across eukaryotes, only three studies have examined the methylation status of entire marsupial chromosomes [2–4] and mostly in the context of X chromosome inactivation, part of the dosage compensation mechanism to equalise gene expression between males with one X chromosome and females with two. Unfortunately, none of these studies have examined the methylation status in both male and female cells from the same species and they have also not provided a detailed analysis of the distribution of methylation on autosomes and the similarities/differences between species.

**Fig. 1:**
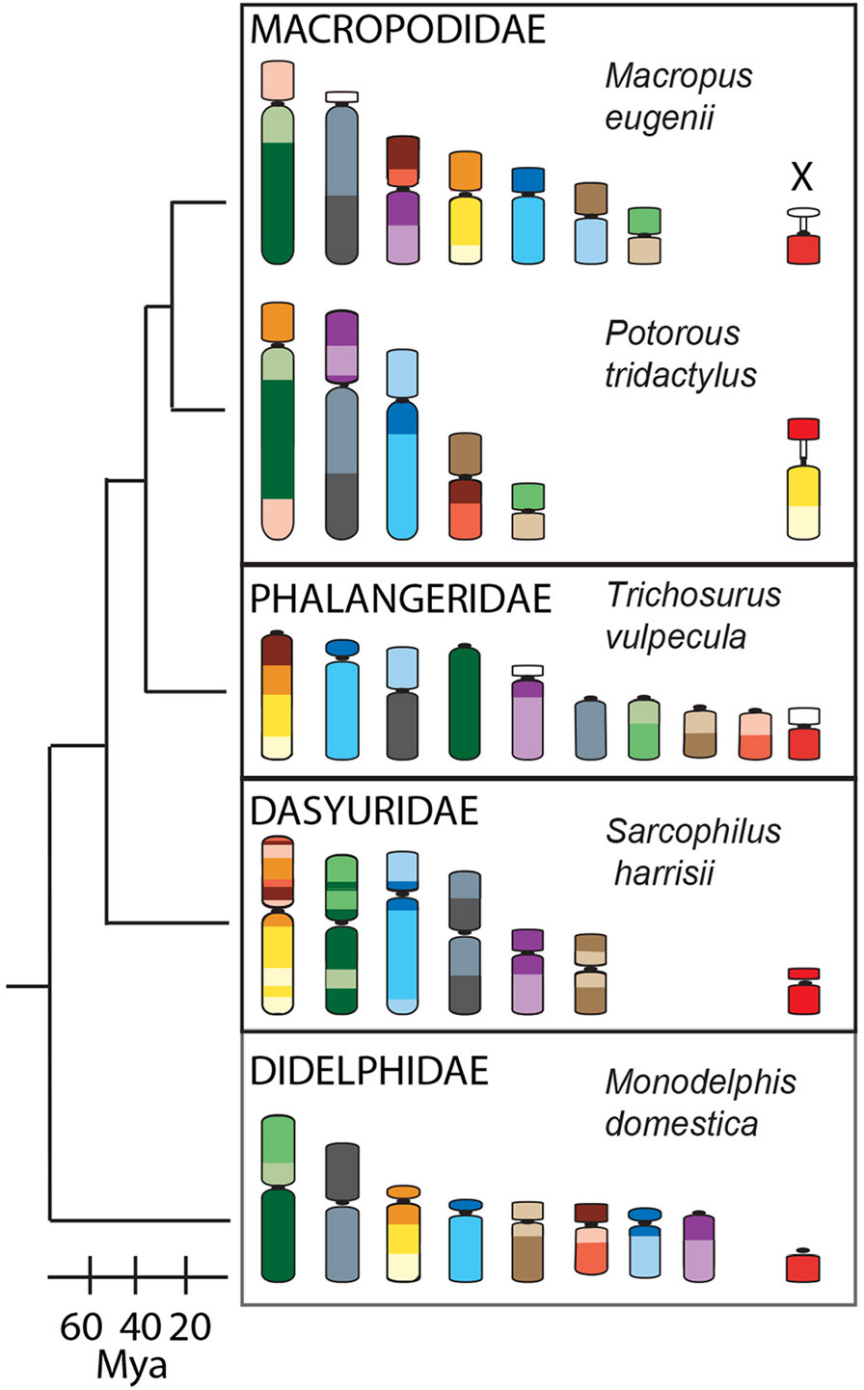
Marsupial phylogeny showing relationship of the species used in this and previous studies. Female haploid chromosome complements are colour-coded to indicate the homology between species

The Tasmanian devil (*Sarcophilus harrisii*) is an interesting species in which to study methylation patterns on chromosomes. Firstly, it has a 2n = 14 karyotype similar to the predicted ancestral marsupial karyotype [5] (Fig. 1). Secondly, devil chromosomes display a remarkable telomere length dimorphism where one homologue of each chromosome has short telomeres while the other has long, with this dimorphism proposed to be due to a parent-of-origin effect and possibly epigenetically regulated [6]. Finally, and most importantly, the devil population has been decimated by a transmissible tumour known as devil facial tumour disease (DFTD). Some chromosomes in DFTD have undergone extensive rearrangement [7, 8], providing an opportunity to examine the effect of genome rearrangement on chromosome methylation. Changes in DNA methylation are common in tumours, with hypermethylation of promoter regions leading to the silencing of tumour suppressor genes and hypomethylation, particularly of repeat sequences, being associated with genome instability. The cytogenetic monitoring of DFT samples collected from 2005 to 2012 [9] and detailed molecular cytogenetic analysis performed on the different DFT strains [8], enables chromosome rearrangements to be correlated with changes in methylation status as the tumour evolves.

### Methylation of marsupial chromosomes

Methylation studies on marsupial chromosomes have been performed on several Australian and one American species. Figure 1 shows the phylogenetic relationship of species used in previous studies and their chromosome complements. O’Neill et al. [3] used a nick translation technique, which detected unmethylated DNA, on two members of the Macropodidae family, the tammar wallaby (*Macropus eugenii*) and the swamp wallaby (*Wallabia bicolor*). Centromeric/pericentric regions of chromosomes from both macropodid species were more methylated than the rest of the chromosome for each of the autosomes, a feature that was not detected for species from other families [2]. No attention was drawn to the sex chromosomes in this study and indirect measurement afforded by this technique made it difficult to assess the methylation status of the sex chromosomes. The other two marsupial chromosome methylation studies focused primarily on the X chromosome. The consensus pattern of methylation status of the X chromosomes in female Australian marsupials examined (brushtail possum - *Trichosurus vulpecula*; potoroo – *Potorous tridactylus;* wallaroo– *Macropus robustus*) is that the inactive X is hypomethylated compared to the active X and autosomes [2, 4]. Unfortunately, there is no published data on the methylation status for males of these species. Conversely, for the South American opossum (*Monodelphis domestica*), only male metaphase chromosomes have been examined for methylation status, which demonstrated a methylated X chromosome and unmethylated Y chromosome [2]. The patchy data therefore warrant a more thorough investigation of methylation on autosomes and sex chromosomes in both sexes for representative marsupials.

### Devil facial tumour disease chromosomes

**Fig. 2:**
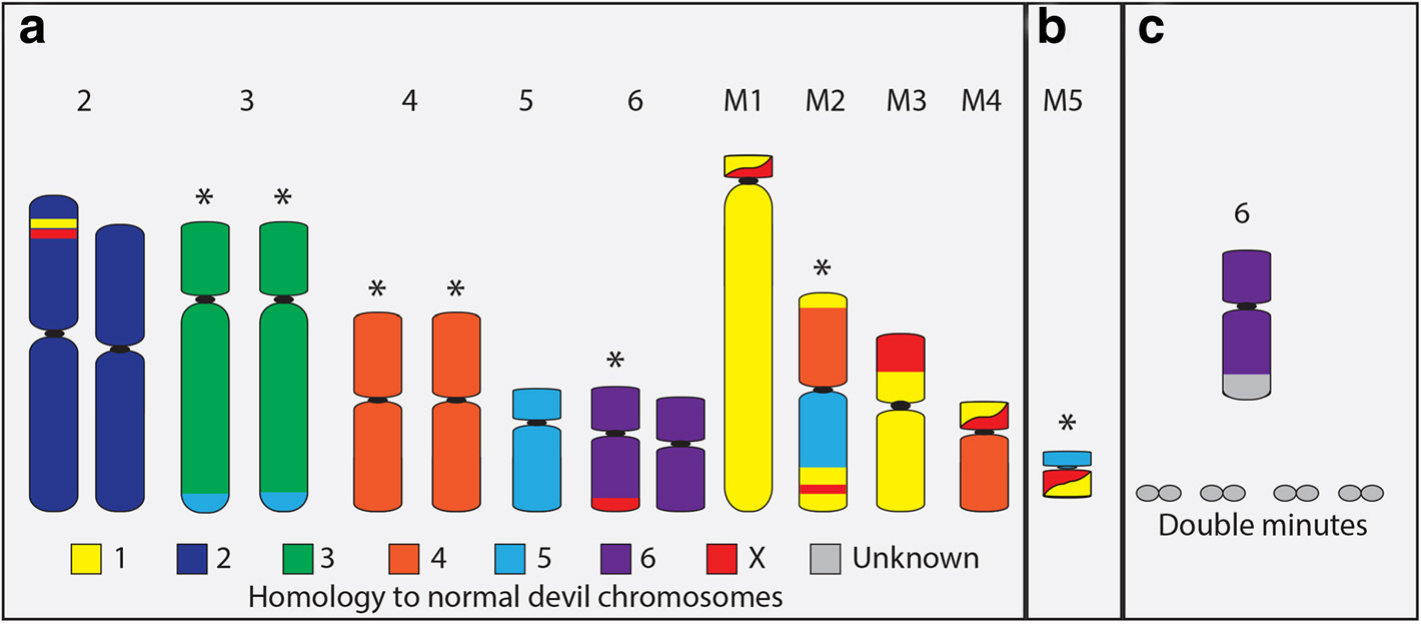
DFT chromosomes and their homology to normal devil chromosomes. **a** Chromosomes present in all strains. **b** M5 is present in strains 2 – 4. **c** Chromosome 6 in strain 4 has had an addition to the long arm, the origin of which is currently unknown. Variable numbers of double minutes are also present in strain 4. * denotes chromosomes with variations within and between strains [8]

Evidence from cytogenetic and sequencing analyses indicate that the original DFTD tumour was derived from a Schwann cell of a female devil and this tumour has since spread through the population [7, 8, 10, 11]. Transmission of the tumour appears to occur by healthy devils biting into the tumours of infected devils, with biting being a part of normal social interaction [12]. A notable feature of this unusual tumour includes its remarkable karyotypic stability over time. After an initial major genome restructuring, presumed to have resulted from a chromothripsis event, DFT chromosomes have undergone very few changes given the number of cell divisions the tumour would have experienced since its formation [8, 9]. The first reported DFTD karyotypes consisted of 13 chromosomes with both homologues of chromosomes 2, 3, 4 and 6 as well as one homologue of chromosome 5 recognisable [7]. Material from chromosomes 1, 4, 5 and X was rearranged to form four marker chromosomes (M1 – M4) [8] (Fig. 2a). There are now four reported karyotypic strains of the disease [9]. Strains 2, 3 and 4 have an additional small marker chromosome (M5) (Fig. 2b), rearrangements and/or deletions on the short arm of chromosome 3 (strains 2 and 3), a presumed addition to one homologue of chromosome 6 (strain 4) and the presence of double minutes (strain 4) (Fig. 2c). It has been suggested that either strain 1 or 2 appear closest to the original DFTD cell line, with strains 3 and 4 most likely being offshoots of strain 2 [9]. Tetraploid versions have been observed for each of the strains [9].

Changes in DNA methylation have been observed in DFTs [13]. These differences do not correspond to karytopic strains but are instead correlated with time. Using the methylation sensitive amplified fragment length polymorphism technique, methylation levels for full and hemi-methylation were measured in the years 2005 to 2007 and 2010. A decrease in methylation was caused by a significant loss of hemi-methylated sites over time. Full methylation increased over time. No significant differences in any methylation levels were detected across all four strains. Overall demethylation was attributed to increasing upregulation of *MBD2* and *MBD4* genes associated with active DNA demethylation [13]. However, the technique used to detect these methylation differences is unable to determine whether these changes in the extent of DNA methylation are experienced globally across the DFT genome or localized to specific regions. In human cancer genomes, a decrease in methylation is mostly targeted to specific genomic regions, such as repetitive sequences and decreased methylation of repeat sequences has been associated with chromosome instability in certain cancers [14].

Given the detailed cytogenetic analysis that has been performed on karyotypic strains of DFTD and cytogenetic monitoring of tumour samples collected until 2013, using an approach able to show methylation status on a chromosome would enable chromosome rearrangements to be correlated with methylation status. One approach to determining methylation status at a chromosome level is to use immunofluorescent staining with an antibody to 5-methylcytosine. This approach allows cells and chromosomes to be observed individually, also permitting heterogeneity within a sample to be detected.

We performed immunofluorescent staining on male and female fibroblast cells from three marsupial species and DFT chromosomes from samples obtained from 2005 to 2013 and different DFT strains. We show species-specific methylation patterns on metaphase chromosomes prepared from marsupial fibroblast cells, distinct differences in the level of methylation on sex chromosomes, an association of X chromosome material with hypermethylation on DFTD chromosomes, and stability of global methylation patterns on DFTD chromosomes over time.

## Results and discussion

We examined the distribution of DNA cytosine methylation on male and female chromosomes of three distantly related, model marsupial species [15] using an immunofluorescent staining approach. Methylation status of male tammar wallaby [3] and male opossum fibroblasts [2] has previously been determined and were therefore used in this study as controls for the technique. We extended these experiments to both sexes for each species as well as male and female devil fibroblasts to compare methylation patterns between species for autosomes and sex chromosomes. We then examined the methylation status of the rearranged DFT genome, comparing the methylation patterns in different karyotypic strains as well as samples of the same strains taken in different years.

### Comparison of DNA methylation patterns on marsupial autosomes

**Fig. 3:**
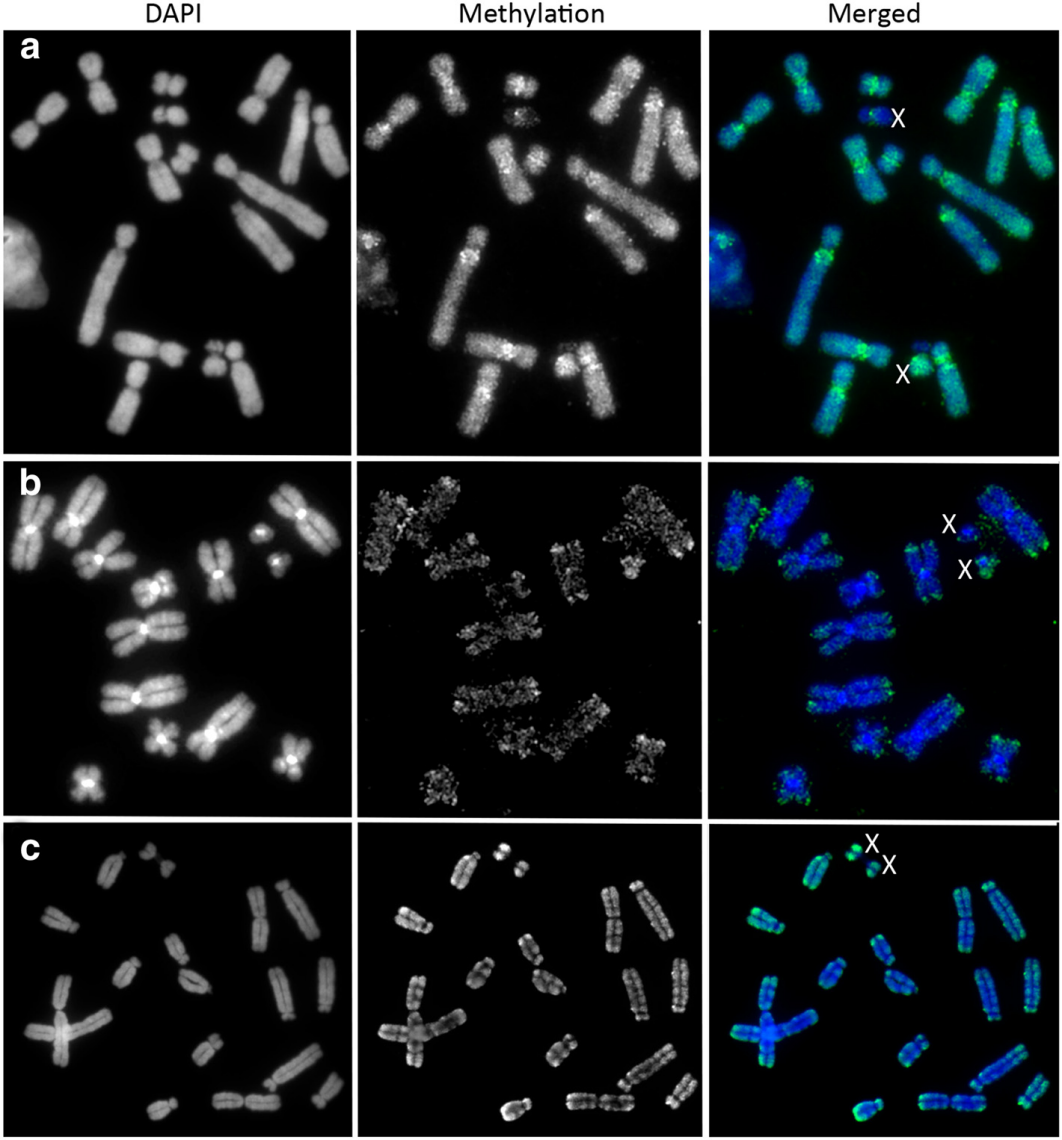
Methylation status of metaphase chromosomes from **a** tammar wallaby, **b** devil and **c** opossum female fibroblast cells. Images for DAPI and 5-methylcytosine are shown separately as well as merged. X chromosomes are indicated

**Fig. 4:**
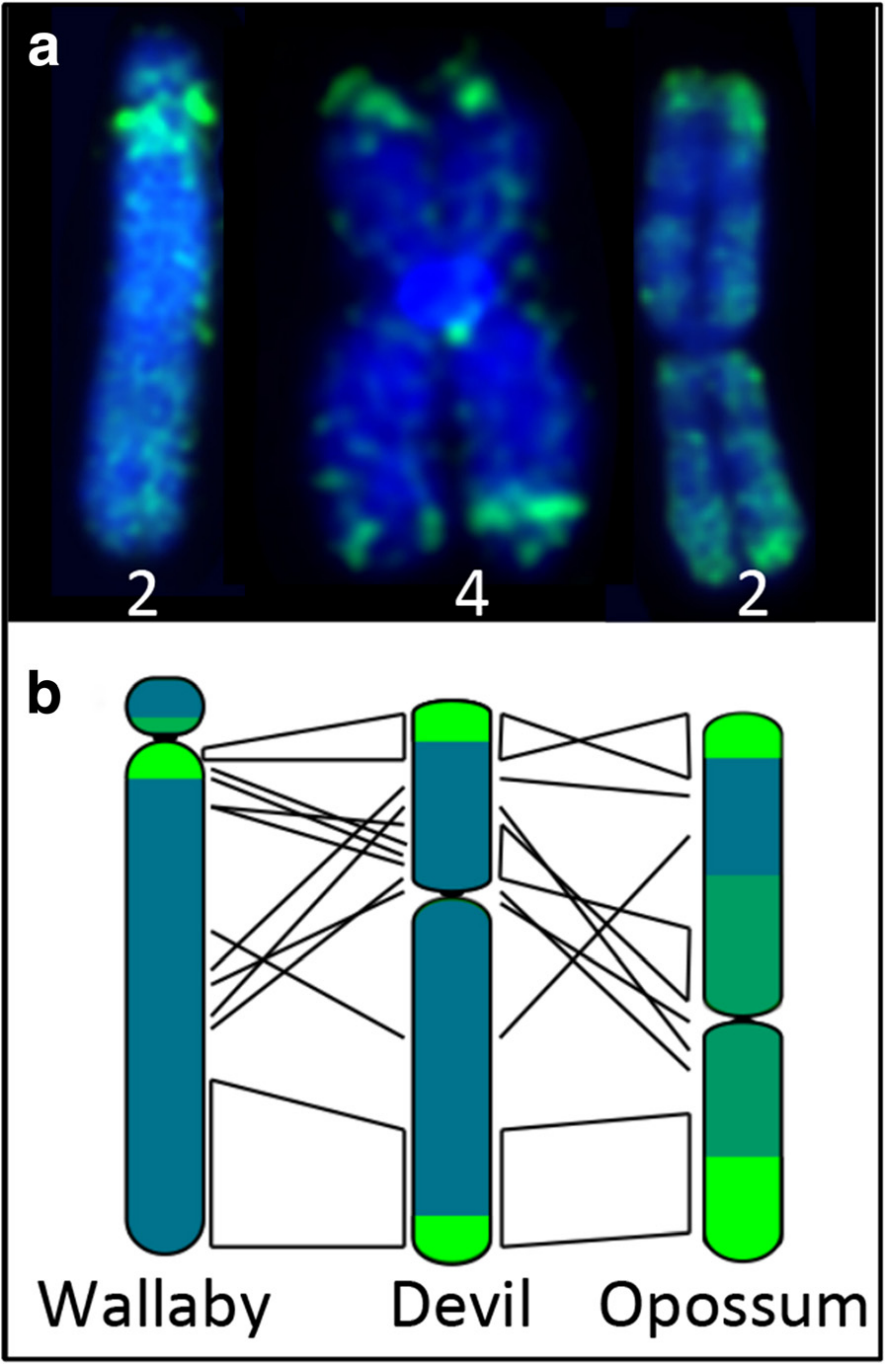
Between species comparisons of global methylation for a chromosome with the same gene content. **a** Immunostained chromosomes of tammar wallaby chromosome 2, devil chromosome 4 and opossum chromosome 2. **b** Schematic depicting the gene rearrangements between species and their methylation status indicated in green

All tammar wallaby autosomes had intense staining for the anti-5-methylcytosine antibody in the pericentric regions and more moderate staining along the rest of the chromosome (Fig. 3a). This was consistent with the pattern previously described using a nick translation technique [3]. The telomeric/subtelomeric regions of chromosomes 1 and 3 were consistently stained, although less intensely than that in the pericentric region. Devil fibroblasts displayed strong staining for 5-methylcytosine in telomeric regions, and faint levels of staining on remaining autosomal regions (Fig. 3b). There were no detectable differences in intensity or extent of methylation in telomeric regions of homologous chromosomes. Therefore, the intensity of methylation staining using the immunofluorescence technique does not appear to correspond to the telomere length dimorphism observed in this species [6]. The opossum also had strong staining of telomeric regions on autosomes, and a lack of methylation in centromeric regions. The rest of the autosomal regions had more moderate levels of methylation (Fig. 3c). Comparisons of methylation patterns between species for autosomes with the same gene content demonstrated a methylation profile independent of gene content. This is indicated in Fig. 4 where a comparison of devil chromosome 4 with its homologue in the tammar wallaby (chromosome 2) and opossum (chromosome 2) shows that the conserved region on the distal region of the long arm of each of these chromosomes has a different methylation pattern. However, most intense methylation staining was apparent in heterochromatic regions (centromeres or telomeres).

The intense staining in pericentric regions of tammar wallaby chromosomes is intriguing given the rapid karyotypic evolution within the family Macropodidae. Marsupials are renowned for their well-conserved karyotypes except within the family Macropodidae, where there is considerable karyotypic diversity with diploid numbers ranging from the smallest (2n = 10,11 in *Wallabia bicolor*) to the largest (2n = 32 in *Aepyprymnus rufescens*) amongst marsupials [16]. Most rearrangements involve centromeres, either through centric fusions, fissions, inversions, translocations or shifts [17–19]. Chromosome rearrangements in macropodid species hybrids also predominantly involve centromeres [20]. The demethylation of kangaroo endogenous retrovirus (KERV) present in the centromeric region and its subsequent amplification were associated with the chromosome remodeling in a *Macropus rufogriseus* x *M. eugenii* hybrid [3]. KERV is located at the centromeres and small part of the pericentric region of all tammar wallaby chromosomes, and to a lesser extent at regions corresponding to latent centromeres [19, 21]. Some of these latent centromere regions, such as one on the distal end of the long arm of chromosome 3, correspond to regions stained for 5’methylcytosine (Additional file 1). Thus, it would appear that methylation of the centromeric/pericentric regions is important for genome stability in macropodid species. Although the presence of KERV has been determined in the devil and opossum [19], the location of KERV in their genomes, its copy number and expression is yet to be determined. The hypomethylation of centromeric regions in devil and opossum would make it interesting to examine KERV using a sequencing approach in all three species.

The vertebrate telomere repeat sequence (TTAGGG) lacks the CpG dinucleotide required for DNA methylation. However, telomeric repeats of Australian marsupials, including those of the devil, are disrupted by other DNA sequences [6, 13, 22] which could contain the CpG dinucleotides required for methylation. Whether these additional DNA sequences also exist in the opossum is unknown but telomeres of another member from the Didelphidae family, the North American opossum (*Didelphis virginiana*) seems to lack these additional sequences [22]. Hence, the hypermethylation observed in the telomeric regions on devil and opossum chromosomes may not correspond to methylation of sequence within the telomeres themselves but to methylation of subtelomeric sequences. Subtelomeric regions in humans and mice are GC rich and hypermethylated [23–25], with methylation of subtelomeric regions implicated in repressing DNA recombination of repetitive sequences in these regions and acting as an indirect regulator of telomere length [24]. Methylation of these regions in two distantly related marsupials may mean that a similar mechanism is present in at least some marsupials. In the tammar wallaby and other macropodid species, the telomeric repeat probe hybridises much more strongly to centromeres with little or no hybridisation detected at telomeres [6, 26]. It has been suggested that the TTAGGG repeat has been incorporated into the centromeric satellite sequence [26]. One interesting observation is the overlap of telomere repeat hybridisation with methylation staining on tammar wallaby chromosomes. Again, it would be useful to use a targeted sequencing approach for sequences from these regions.

### Methylation of marsupial sex chromosomes

Although the methylation status of the X chromosomes in marsupial fibroblasts has been previously reported [2, 4], no other study has examined X chromosomes in both sexes from the same species. We have rectified this situation by examining the methylation status of X chromosomes in both sexes for three species.

**Fig. 5:**
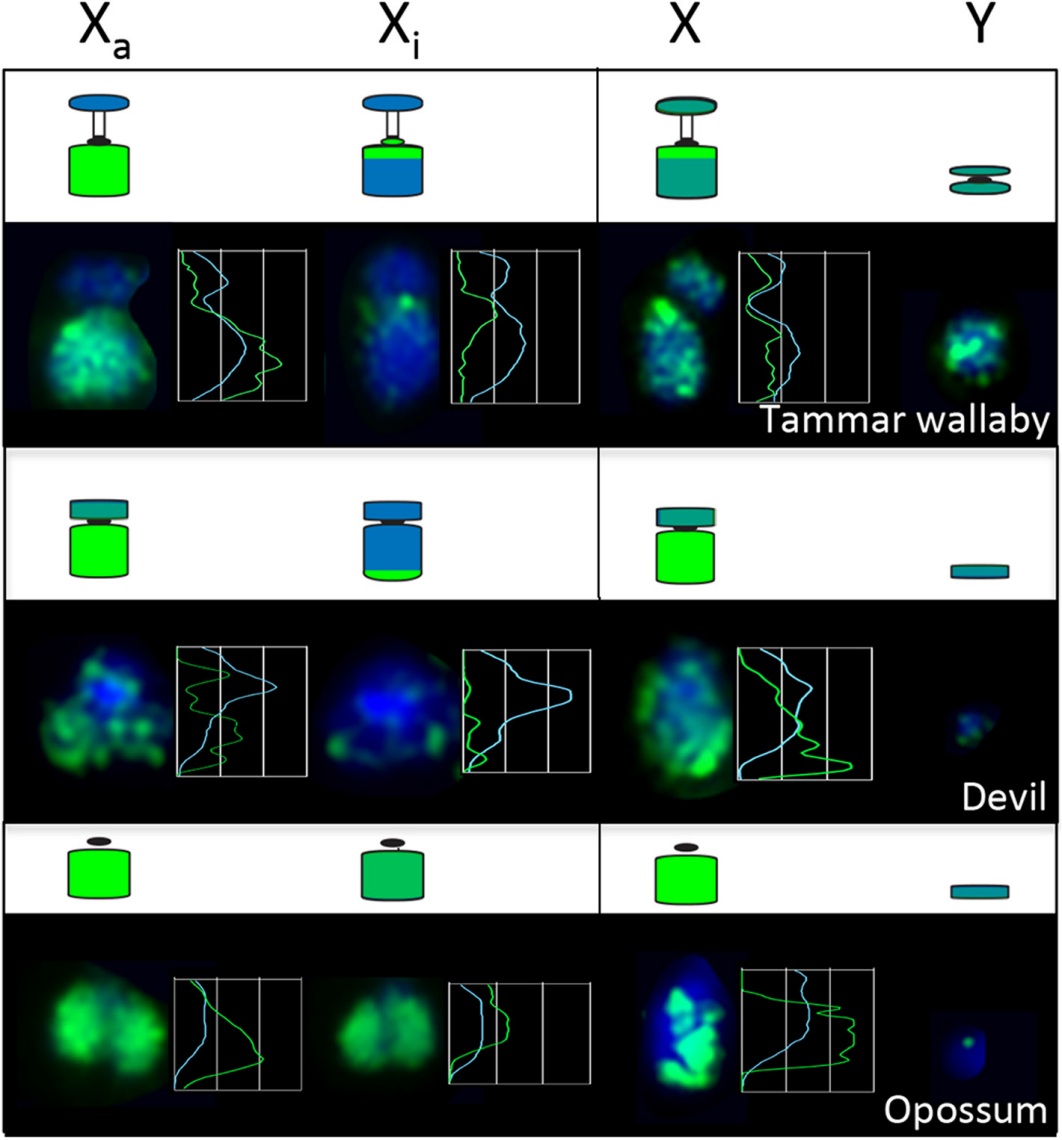
Methylation status of marsupial sex chromosomes. X_a_ and X_i_ denote the active and inactive X chromosomes in females respectively. Line scans indicate the distribution of DNA methylation (green) and DAPI staining (blue) along the X chromosomes

In female tammar wallaby fibroblast cells, the long arm of one X chromosome was hypermethylated compared to the other (Fig. 5). The short arm was hypomethylated on both X chromosomes. In males, both the short and long arms of the X chromosome were hypermethylated. Similar to the tammar wallaby, one devil X in female fibroblasts was hypermethylated compared to the other one, which only had a small amount of staining on the long arm (Fig. 5). In male fibroblasts, the X chromosome was likewise hypermethylated. In contrast, both X chromosomes in female *M. domestica* were methylated, although one more so than the other (Fig. 5) and the male cells possessed a hypermethylated X chromosome. Importantly, the methylation patterns observed for male wallaby and opossum chromosomes were consistent with those observed in the previous studies [2, 3]. Evidence suggests that the hypomethylated X in these species corresponds to the inactive X as this has been demonstrated previously using sequential staining with the repressive mark H3K9me3 associated with the inactive X followed by staining for 5-methylcytosine in the brushtail possum [2]. This means that the active X is hypermethylated in line with the hypermethylation of the single active X chromosome in males.

Hypomethylation of the inactive X is not restricted to marsupials but has also been observed for human fibroblasts [27, 28]. It has been perplexing that, at the cytogenetic level, the human inactive X is hypomethylated when hypermethylation of gene promoter regions has been associated with gene silencing on the inactive X in eutherians mammals [29, 30]. Methylation of 5’ CpG sites on the inactive X is lacking for marsupial X-borne genes, questioning the role of methylation in the X inactivation mechanism in marsupials [31–34]. Higher levels of methylation have been reported for the active compared to inactive X in gene bodies [30] and intergenic regions in humans [35] and for gene bodies of two X-borne genes (*HPRT1* and *G6PD*) in marsupials [33, 36], which would be consistent with the cytogenetic observations. Nonetheless, a more thorough examination of gene body methylation differences in marsupials is warranted. Although the function of gene body methylation is currently poorly understood, it is conserved in many species of eukaryotes [37, 38], suggesting that it plays an important biological function. Gene body methylation has some correlation with gene expression, as moderately expressed genes are more likely to be methylated than those that have very low or high levels of expression [37–40]. Thus, demethylation of the inactive X may play a role in the X chromosome inactivation mechanism.

The hypermethylation of the tammar wallaby Y chromosome was in contrast to the near absence of detectable methylation of the tiny devil and opossum Y chromosomes (Fig. 5). This methylation difference is most likely due to the large heterochromatic region on the tammar wallaby Y chromosome that shares homology with the heterochromatic short arm of the tammar wallaby X chromosome [41]. Similarly, in human fibroblasts the heterochromatic region on the long arm of the Y chromosome, a region rich in repetitive DNA, stains strongly for 5-methylcytosine whereas the mouse and lemur Y chromosomes are predominantly hypomethylated with the exception of the telomeric regions [27].

### Methylation patterns on DFT chromosomes

**Fig. 6:**
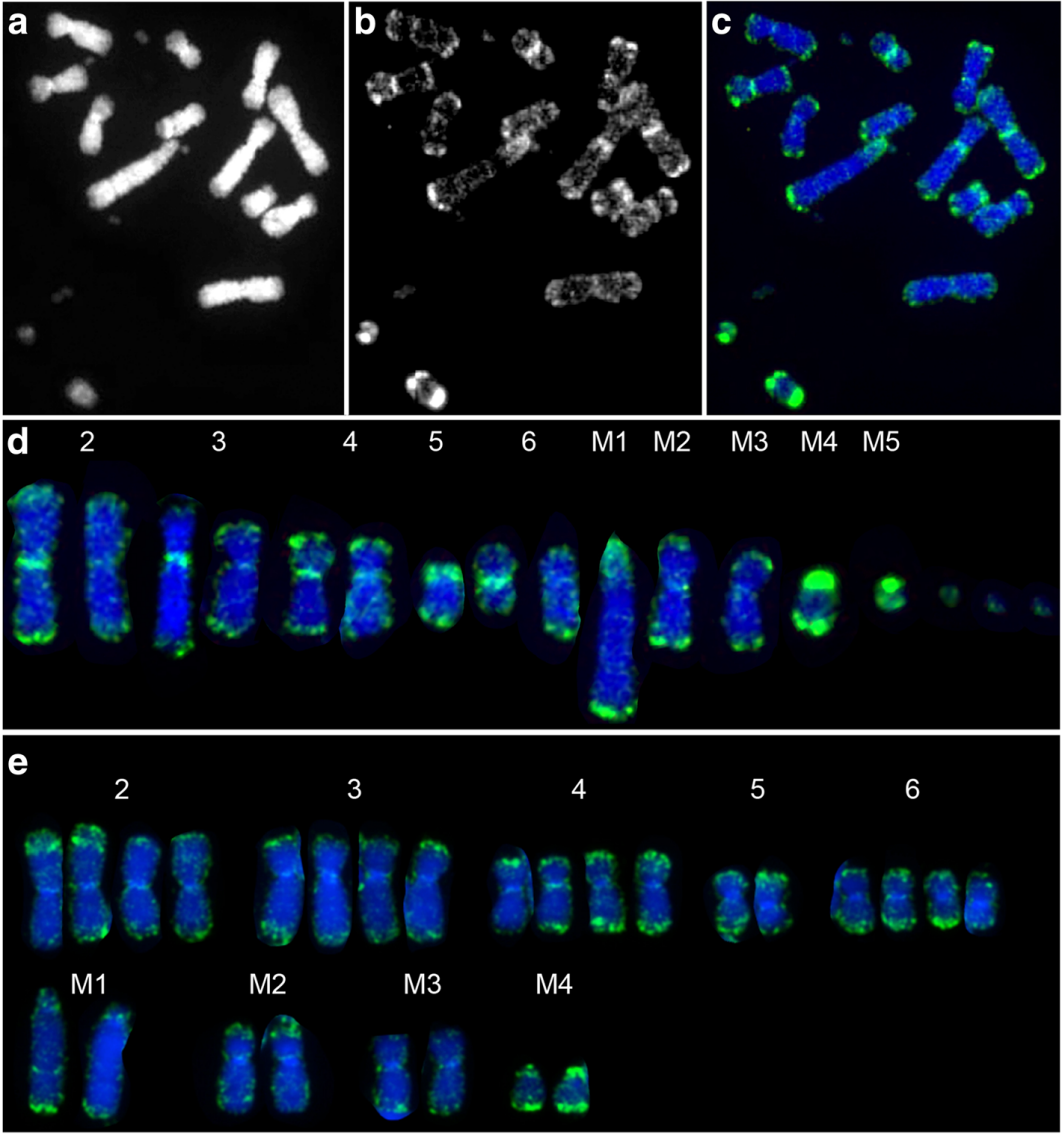
Methylation staining on DFT chromosomes. **a** DAPI image, **b** methylation staining and **c** merged image showing methylation staining on strain 4 chromosomes. **d** karyotype of chromosomes depicted in images a-c. **e** karyotype of a tetraploid strain 1 tumour

Similar to chromosomes from devil fibroblast, all DFTD chromosomes, including the rearranged marker chromosomes, displayed strong telomeric region methylation, and little interstitial methylation (Fig. 6a-d). It is particularly interesting that the marker chromosomes, for which the original telomeres would have been lost during rearrangement, have reinstated methylation of telomeric regions. Methylation of these regions may therefore be important for telomere length maintenance and tumour persistence.

Staining of the centromeric region was observed in all samples, albeit at varying intensities. The short arm of the marker chromosome 1 was hypermethylated, extending into the pericentric region of the long arm. There was also a consistent, narrow band of methylation two thirds of the way down the long arm of M1. The most hypermethylated region in the DFT karyotype was on marker chromosome 4, which was characterised by very intense methylation at both telomeric ends, but particularly on the short arm (Fig. 6). Marker chromosome 5 (present in all strains except strain 1) displayed intense methylation skewed to the short arm of the chromosome. Chromosomes prepared from different cultures established from the same tumour sample ensured experimental consistency (Strain 1 - #06/2617 and #06/2887; strain 4 - #07/0192). Chromosome tetraploidy was observed in one strain 1 sample examined but the broad methylation patterns remained unaffected (Fig. 6e).

**Fig. 7:**
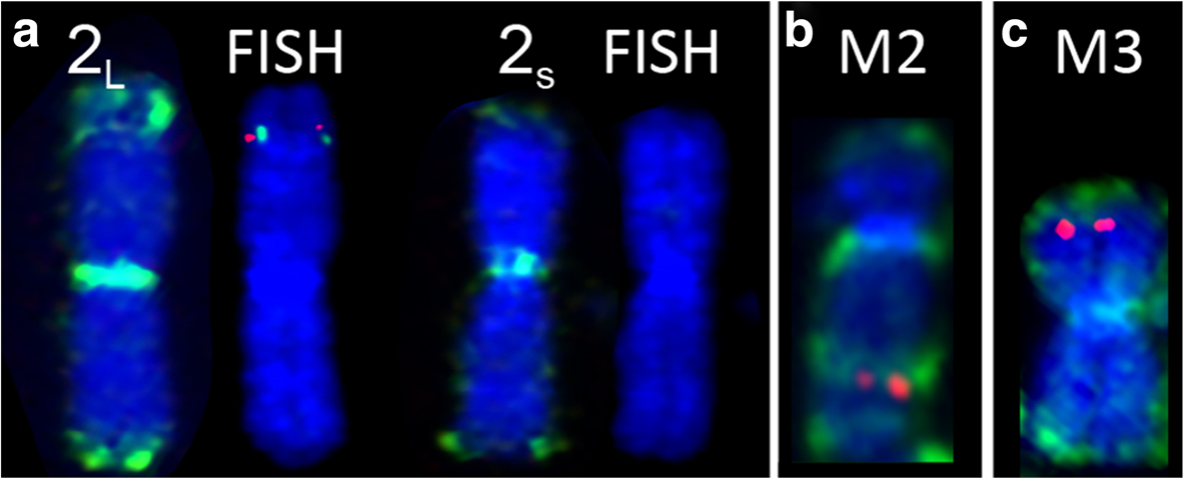
Colocalisation of X-borne genes and hypermethylated regions. **a** The region containing X-borne genes (*PLP –* green; *SRPX2 –* red) overlaps with the extended hypermethylated region on the longer homologue of chromosome 2 (2_L_). The hypermethyated region and X-borne genes are absent from the shorter chromosome 2 homologue (2_s_). **b** *HEPH* (red) and methylation staining (green) on marker chromosome 2. **c** *ARHGEF6* and methylation staining (green) on marker chromosome 3

One chromosome 2 homologue had an extended methylation range on one of its arms, which was observed across all strains and time periods. Based on chromosome measurements, this increased methylation was always present on the longer chromosome 2 homologue, which has been shown to have an added region consisting of DNA material from chromosomes 1 and X [8]. We performed fluorescence *in situ* hybridisation on the same metaphase spreads that had been stained for 5-methylcytosine, with two X chromosome genes (*PLP* and *SRPX*) mapping to this region on DFTD chromosomes, demonstrating that the extended region of methylation overlaps with the translocated genes (Fig. 7a). Similarly, an additional region of methylation was observed on M2 in the region where X-borne gene *HEPH* is located (Fig. 7b). The hypermethylated M4 and M5 chromosomes have also been previously shown to harbour material from the X chromosome [8]. Interestingly, the short arm of M3, which also shares homology with the X chromosome, was not hypermethylated beyond the telomeric region (Fig. 7c). The proposed chromothripsis event is thought to have resulted in the shattering of one homologue each of chromosomes 1 and X, distributing X material across chromosomes 2, 6, M1, M2 and M4. The short arm of chromosome M4 appears to have been duplicated to form the long arm of M5. The X chromosome material on M3 corresponds to almost an entire, yet rearranged, X chromosome [8]. We posit, based on the hypermethylation of regions corresponding to X chromosome fragments on chromosomes 2, 6, M1, M2 and M4, that the active X was shattered in the chromothripsis event and the hypomethylated X chromosome material on M3 corresponds to the inactive X.

In marsupials, it is the paternally derived X chromosome that is preferentially inactivated and the maternal X is always active [34, 42], meaning that if the active X was indeed the one that was shattered, it was of maternal origin. Interestingly, devils have a telomere length dimorphism where the one haploid set of chromosomes has long telomeres and the other set has short telomeres [6]. Based on the observation in males that the Y chromosome always had long telomeres, it was proposed that telomere length in devils is subject to parental control, with the haploid set of chromosomes with long telomeres being paternally derived and the set with short telomeres being maternally derived [6]. Thus, it is curious that the maternally derived X chromosomes is potentially the one fragmented in DFTD. Were the telomeres of the maternal X and chromosome 1 lost, due to their short length to begin with, resulting in the formation of breakage-fusion-bridge cycles and extensive rearrangement?

We also detected methylation patterns associated with karyotypic differences between strains. For example, a rearrangement in a chromosome 6 homologue of strain 4 [9] was accompanied by strong epigenetic methylation at the rearrangement (Additional file 2). Double minutes present only in strain 4 [9] were only faintly stained with DAPI suggesting that they are GC-rich and yet only faintly stained for 5-methycytosine (Fig. 6a-d). These extrachromosomal DNA fragments typically represent amplified genes in tumours [43] in which hypomethylation of CpG islands is proposed to play a role in their formation [44], perhaps explaining their low level of methylation.

No consistent changes in DNA methylation over time were observed within DFT cells at the chromosome levels (Additional file 3), contrary to expectations based on the study by Uvjari et al. [13]. This may be attributed to hypomethylation over time occurring at hemi-methylated rather than fully methylated sites as well as the less quantitative nature of the immunofluorescent technique used here. However, the conservation of the broad methylation pattern over time may be a contributing factor to the genomic stability the DFT has demonstrated.

## Conclusions

Immunostaining for 5-methylcytosine on chromosomes from three distantly related marsupials demonstrated species-specific methylation patterns yet a conserved hypermethylation of one X chromosome compared to the other in all three species. Fragments from one X chromosome in DFTs were hypermethylated, suggesting that X chromosome shattered in DFTD corresponds to the active X.

Methylation patterns on DFT chromosomes have remained stable over time. Only minor differences between strains were detected, which correspond to karyotypic differences. The lack of global methylation changes may have attributed to the relatively stable nature of this unusual tumour.

## Methods

### Metaphase chromosome preparations

Fibroblast cell lines used were previously established at the Australian National University with samples collected under approval from the Australian National University Animal Experimentation Ethics Committee (AEECP R.CG.08.03 and AEECP R.CG.11.06). Metaphase chromosomes from male and female devil (2*n* = 14), tammar wallaby (2*n* = 16) and opossum (2*n* = 18) fibroblasts were prepared from cell cultures and dropped onto slides as previously described by Alsop et al. [45]. DFT samples were collected under approval of the Tasmanian Department of Primary Industries and Water Animal Ethics Committee. Metaphase chromosomes were prepared by the Department of Primary Industries, Parks, Water and the Environment as described in Pearse et al. [9], and sent to the University of Canberra on slides. Table 1 lists the details of the DFT samples used in this study.

**Table 1:** DFT samples used in this study

Year	Ascession No.	Location	Strain
2005	05/2569	Fentonbury	2
2006	06/2617^a^	Forestier	3
2006	06/2173	Wisedale	1 (tetraploid)
2006	06/2887^a^	St Mary’s	1
2007	07/0192^a^	East Coast	4
2007	07/0817	Mole Creek	2
2008	08/2215	Mt William	2
2008	08/3329	Hampshire	1 (tetraploid)
2009	09/2013	Mt Pleasant	?
2009	09/1466	Forestier	3
2011	11/3917	Hamilton	3
2011	11/2887	St Mary’s	1
2011	11/3178	Coles Bay	4
2012	12/0624	Kempton	2
2012	12/0652	Kempton	2
2012	12/0760	West Pencil Pine	1
2013	13/0794	Takone	2

^a^denotes chromosomes prepared from different cultures established from the same sample

### Immunofluorescent detection of DNA methylation

Slides were dehydrated through an ethanol series (70, 90 and 100 % v/v) and then aged overnight at 37 °C. Metaphase chromosomes were denatured in 70 % formamide (v/v) in phosphate buffered saline (PBS: 137 mM NaCl, 2.7 mM KCl, 10 mM NA_2_HPO_4_, 2 mM 2.4 KH_2_PO_4_,) at 70 °C for 1 min 40 (fibroblasts) or 30 s (DFTD). The slides were then quenched in ice cold 70 % (v/v) ethanol for 5 min, dehydrated in 90 % (v/v) and 100 % (v/v) ethanol for 3 min each and allowed to air dry. Slides were rinsed in PBS-T (PBS with 0.03 % (v/v) Polysorbate 20) for 3 min before blocking for 20 min in 1 % (w/v) Bovine Serum Albumin in PBS-T. The anti-5-methylcytosine (Clone 10G4) (Zymo Research, Irvine, CA, USA) primary antibody was diluted 1:200 in PBS-T and 200 μl of was added to each slide and incubated in a humid chamber for 1 h at 37 °C. Slides were washed twice for 5 min in PBS-T before adding 200 μl of the secondary antibody (anti-mouse Cy3) diluted 1:500 in PBS-T to the slide and incubating for 1 h in a humid chamber at 37 °C. Wash steps were repeated as before and then the chromosomes were fixed in 4 % (w/v) paraformaldehyde in PBS for 15 min at room temperature. The fixed slides were washed in PBS-T three times for 3 min per wash, left to air dry before mounting with DAPI (4’,6-diamidino-2-phenylindole) in Vectashield (Vector Laboratories Inc., Burlingame, CA, USA). Slides were examined using a Zeiss Axiocam MRm Scope A1 epifluorescence microscope (Carl Zeiss Ltd, Cambridge, UK). Images were captured using an AxioCam Mrm Rev.3 CCD camera (Carl Zeiss Ltd) and ISIS Fluorescence Imaging System software version 5.4.11 (Metasystems, Newton, MA, USA). For each experiments, 24–64 metaphase spreads were captured and analysed. Line scans of individual chromosomes were performed using the ISIS software. Chromosomes were distinguished from each other based on their morphology and size in DAPI images.

#### Fluorescent in situ hybridisation (FISH)

DNA for BAC clones from the VMRC50 library (25 L16 - *HEPH*, 34 L9 – *SRPX2*, 63 J13 - *PLP*, 143E17-*ARHGEF6*) was extracted using the PhasePrep BAC DNA kits (Sigma-Aldrich Pty Ltd, Castle Hill, NSW, Australia) according to the manufacturer’s instructions. The DNA was fluorescently labelled with either SpectrumGreen or Spectrum Orange dUTP (Abbott Molecular Inc., Des Plaines, IL, USA) as previously described [45]. A slide previously subjected to stain for methylation was rinsed in 2× saline sodium citrate (SSC) buffer (0.3 M NaCl, 0.03 M sodium citrate, pH7) and dehydrated through an ethanol series (70, 90 and 100 % v/v) before being hybridised with the fluorescently labelled probes as described by Alsop et al. [45]. The slide was washed to remove any unbound probe and mounted using the same protocol initially used to identify the location of these BACs on normal devil and DFT chromosomes [8]. Fluorescent signals were visualised and captured using the same microscope and imaging software that was used for the methylation staining.

## Additional files

Additional file 1:
**Comparison of KERV distribution and DNA methylation on tammar wallaby chromosome 3.** KERV distribution [19] is indicated in red on the chromosome 3 ideogram and compared to the distribution of methylation (green) on the immunostained chromosome. The line scan indicated the intensity of methylation (green) and DAPI staining (blue). (TIFF 345 kb) Additional file 2:
**Methylation pattern on chromosome 6 from Strain 4.** The DAPI image is shown to the left of the immunostained image. Hypermethylation is observed either side of a darker staining DAPI band on the long arm, corresponding to added region. Line scan indicates intensity of methylation staining (green) and DAPI staining (blue). (TIFF 325 kb) Additional file 3:
**Comparison of strain 1 global methylation between samples taken in 2006 and 2012.** (TIFF 766 kb)

## Abbreviations

CCD: Charged-coupled device
DAPI: 4’,6-diamidino-2-phenylindole
FISH: Fluorescence in situ hybridization
DFTD: Devil facial tumour disease
DFT: Devil facial tumour
KERV: Kangaroo endogenous retrovirus
PBS: Phosphate buffered saline
SSC: Saline sodium citrate

## References

[1] Deakin JE, Domaschenz R, Siew Lim P, Ezaz T, Rao S (2014). Comparative epigenomics: an emerging field with breakthrough potential to understand evolution of epigenetic regulation. AIMS Genet.

[2] Rens W, Wallduck MS, Lovell FL, Ferguson-Smith MA, Ferguson-Smith AC (2010). Epigenetic modifications on X chromosomes in marsupial and monotreme mammals and implications for evolution of dosage compensation. Proc Natl Acad Sci U S A.

[3] O’Neill RJ, O’Neill MJ, Graves JA (1998). Undermethylation associated with retroelement activation and chromosome remodelling in an interspecific mammalian hybrid. Nature.

[4] Loebel DA, Johnston PG. Analysis of DNase 1 sensitivity and methylation of active and inactive X chromosomes of kangaroos (*Macropus robustus*) by in situ nick translation. Chromosoma. 1993;102:81–7. 10.1007/BF00356024 8381740

[5] Deakin JE, Delbridge ML, Koina E, Harley N, Alsop AE, Wang C, et al. Reconstruction of the ancestral marsupial karyotype from comparative gene maps. BMC Evol Biol. 2013;13:258. 10.1186/1471-2148-13-258 PMC4222502 24261750

[6] Bender HS, Murchison EP, Pickett HA, Deakin JE, Strong MA, Conlan C, et al. Extreme Telomere Length Dimorphism in the Tasmanian Devil and Related Marsupials Suggests Parental Control of Telomere Length. PLoS ONE. 2012, 7. 10.1371/journal.pone.0046195 PMC3458001 23049977

[7] Pearse A-M, Swift K (2006). Allograft theory: transmission of devil facial-tumour disease. Nature.

[8] Deakin JE, Bender HS, Pearse AM, Rens W, O’Brien PCM, Ferguson-Smith MA, et al. Genomic restructuring in the Tasmanian devil facial tumour: chromosome painting and gene mapping provide clues to evolution of a transmissible tumour. PLoS Genet. 2012;8. 10.1371/journal.pgen.1002483 PMC3280961 22359511

[9] Pearse A-M, Swift K, Hodson P, Hua B, McCallum H, Pyecroft S, et al. Evolution in a transmissible cancer: a study of the chromosomal changes in devil facial tumor (DFT) as it spreads through the wild Tasmanian devil population. Cancer Genet. 2012;205:101–12. 10.1016/j.cancergen.2011.12.001 22469509

[10] Murchison EP, Tovar C, Hsu A, Bender HS, Kheradpour P, Rebbeck CA, et al. The Tasmanian devil transcriptome reveals Schwann cell origins of a clonally transmissible cancer. Science. 2010;327:84–7. 10.1126/science.1180616 PMC2982769 20044575

[11] Murchison EP, Schulz-Trieglaff OB, Ning Z, Alexandrov LB, Bauer MJ, Fu B, et al. Genome sequencing and analysis of the Tasmanian devil and its transmissible cancer. Cell. 2012;148:780–91. 10.1016/j.cell.2011.11.065 PMC3281993 22341448

[12] Hamede RK, Mccallum H, Jones M (2013). Biting injuries and transmission of Tasmanian devil facial tumour disease. J Anim Ecol.

[13] Ujvari B, Pearse A, Peck S, Harmsen C, Taylor R, Pyecroft S, et al. Evolution of a contagious cancer : epigenetic variation in Devil Facial Tumour Disease Evolution of a contagious cancer : epigenetic variation in Devil Facial Tumour Disease. Proc R Soc B Biol Sci. 2013;280:20131720. 10.1098/rspb.2012.1720 PMC3574417 23135679

[14] You JS, Jones PA. Cancer genetics and epigenetics: Two sides of the same coin? Cancer Cell. 2012;9–20. 10.1016/j.ccr.2012.06.008 PMC3396881 22789535

[15] Deakin JE (2012). Marsupial genome sequences: providing insight into evolution and disease. Scientifica (Cairo).

[16] Deakin JE, Graves JAM, Rens W (2012). The evolution of marsupial and monotreme chromosomes. Cytogenet Genome Res.

[17] Rofe R (1978). G-banded chromosomes and the evolution of Macropodidae. Aust Mammal.

[18] Hayman D (1989). Marsupial Cytogenetics. Aust J Zool.

[19] Ferreri GC, Liscinsky DM, Mack JA, Eldridge MDB, O’Neill RJ (2005). Retention of latent centromeres in the Mammalian genome. J Hered.

[20] O’Neill RJW, Eldridge MDB, Graves JAM (2001). Chromosome heterozygosity and de novo chromosome rearrangements in mammalian interspecies hybrids. Mamm Genome.

[21] Ferreri GC, Marzelli M, Rens W, O’Neill RJ (2004). A centromere-specific retroviral element associated with breaks of synteny in macropodine marsupials. Cytogenet Genome Res.

[22] Gomes NMV, Ryder OA, Houck ML, Charter SJ, Walker W, Forsyth NR, et al. Comparative biology of mammalian telomeres: hypotheses on ancestral states and the roles of telomeres in longevity determination. Aging Cell. 2011;10:761–8. 10.1111/j.1474-9726.2011.00718.x PMC3387546 21518243

[23] Brock GJR, Charlton J, Bird A (1999). Densely methylated sequences that are preferentially localized at telomere-proximal regions of human chromosomes. Gene.

[24] Gonzalo S, Jaco I, Fraga MF, Chen T, Li E, Esteller M, et al. DNA methyltransferases control telomere length and telomere recombination in mammalian cells. Nat Cell Biol. 2006;8:416–24. 10.1038/ncb1386 16565708

[25] Lister R, Pelizzola M, Dowen RH, Hawkins RD, Hon G, Nery JR, et al. Human DNA methylomes at base resolution show widespread epigenomic differences. Nature. 2009;462:315–22. 10.1038/nature08514 PMC2857523 19829295

[26] Metcalfe CJ, Eldridge MDB, Johnston PG (2004). Mapping the distribution of the telomeric sequence (T2AG 3)n in the Macropodoidea (Marsupialia) by fluorescence in situ hybridization. II. The ancestral 2n = 22 macropodid karyotype. Cytogenet Genome Res.

[27] Bernardino J, Lombard M, Niveleau A, Dutrillaux B (2000). Common methylation characteristics of sex chromosomes in somatic and germ cells from mouse, lemur and human. Chromosom Res.

[28] Viegas-Pequignot E, Dutrillaux B, Thomas G (1988). Inactive X chromosome has the highest concentration of unmethylated Hha I sites. Proc Natl Acad Sci U S A.

[29] Tribioli C, Tamanini F, Patrosso C, Milanesi L, Villa A, Pergolizzi R, et al. Methylation and sequence analysis around Eagl sites: identification of 28 new CpG islands in Xq24-Xq28. Nucelic Acids Res. 1992;20:727–33. 10.1093/nar/20.4.727 PMC312011 1542569

[30] Hellman A, Chess A (2007). Gene body-specific methylation on the active X chromosome. Science.

[31] Loebel DA, Johnston PG (1996). Methylation analysis of a marsupial X-linked CpG island by bisulfite genomic sequencing. Genome Res.

[32] Hornecker JL, Samollow PB, Robinson ES, Vandeberg JL, McCarrey JR (2007). Meiotic sex chromosome inactivation in the marsupial *Monodelphis domestica*. Genesis.

[33] Kaslow DC, Migeon BR (1987). DNA methylation stabilizes X chromosome inactivation in eutherians but not in marsupials: evidence for multistep maintenance of mammalian X dosage compensation. Proc Natl Acad Sci U S A.

[34] Wang X, Douglas KC, VandeBerg JL, Clark AG, Samollow PB (2014). Chromosome-wide profiling of X-chromosome inactivation and epigenetic states in fetal brain and placenta of the opossum, *Monodelphis domestica*. Genome Res.

[35] Cotton AM, Price EM, Jones MJ, Balaton BP, Kobor MS, Brown CJ (2014). Landscape of DNA methylation on the X chromosome reflects CpG density, functional chromatin state and X-chromosome inactivation. Hum Mol Genet.

[36] Chong S, Piper AA (1996). Methylation sensitive protein binding to an intragenic active X-specific methylated region in the *M. robustus* Hprt gene. Somat Cell Mol Genet.

[37] Feng S, Cokus SJ, Zhang X, Chen P-Y, Bostick M, Goll MG, et al. Conservation and divergence of methylation patterning in plants and animals. Proc Natl Acad Sci U S A. 2010;107:8689–94. 10.1073/pnas.1002720107 PMC2889301 20395551

[38] Zemach A, McDaniel IE, Silva P, Zilberman D (2010). Genome-wide evolutionary analysis of eukaryotic DNA methylation. Science.

[39] Zilberman D, Gehring M, Tran RK, Ballinger T, Henikoff S. Genome-wide analysis of *Arabidopsis thaliana* DNA methylation uncovers an interdependence between methylation and transcription. Nat Genet. 2007;39:61–9. 10.1038/ng1929 17128275

[40] Jjingo D, Conley AB, Yi SV, Lunyak VV, Jordan IK (2012). On the presence and role of human gene-body DNA methylation. Oncotarget.

[41] Toder R, Wienberg J, Voullaire L, O’Brien PCM, Maccarone P, Marshall Graves JA (1997). Shared DNA sequences between the X and Y chromosomes in the tammar wallaby - Evidence for independent additions to eutherian and marsupial sex chromosomes. Chromosoma.

[42] Rodríguez-Delgado CL, Waters SA, Waters PD (2014). Paternal X inactivation does not correlate with X chromosome evolutionary strata in marsupials. BMC Evol Biol.

[43] Gebhart E (2005). Double minutes, cytogenetic equivalents of gene amplification, in human neoplasia - a review. Clin Transl Oncol.

[44] Rizwana R, Hahn PJ (1998). CpG islands and double-minute chromosomes. Genomics.

[45] Alsop AE, Miethke P, Rofe R, Koina E, Sankovic N, Deakin JE (2005). Characterizing the chromosomes of the Australian model marsupial *Macropus eugenii* (tammar wallaby). Chromosom Res..

