# Supplementary figures and images for: Global DNA Methylation patterns on marsupial and devil facial tumour chromosomes
Source: Mol Cytogenet. 2015 Oct 1;8:74. doi: 10.1186/s13039-015-0176-x (PMC4591559; doi:10.1186/s13039-015-0176-x)

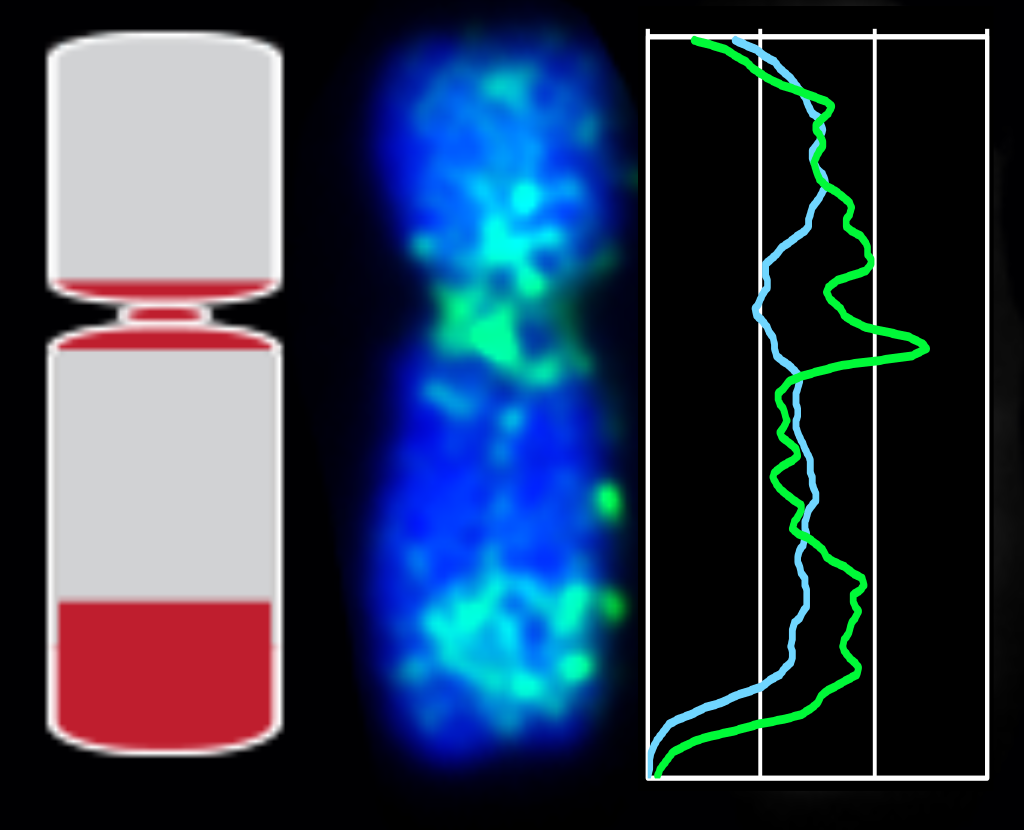

Supplement: Additional file 1: — Comparison of KERV distribution and DNA methylation on tammar wallaby chromosome 3. KERV distribution [19] is indicated in red on the chromosome 3 ideogram and compared to the distribution of methylation (green) on the immunostained chromosome. The line scan indicated the intensity of methylation (green) and DAPI staining (blue). (TIFF 345 kb) [file 13039_2015_176_MOESM1_ESM.tif]

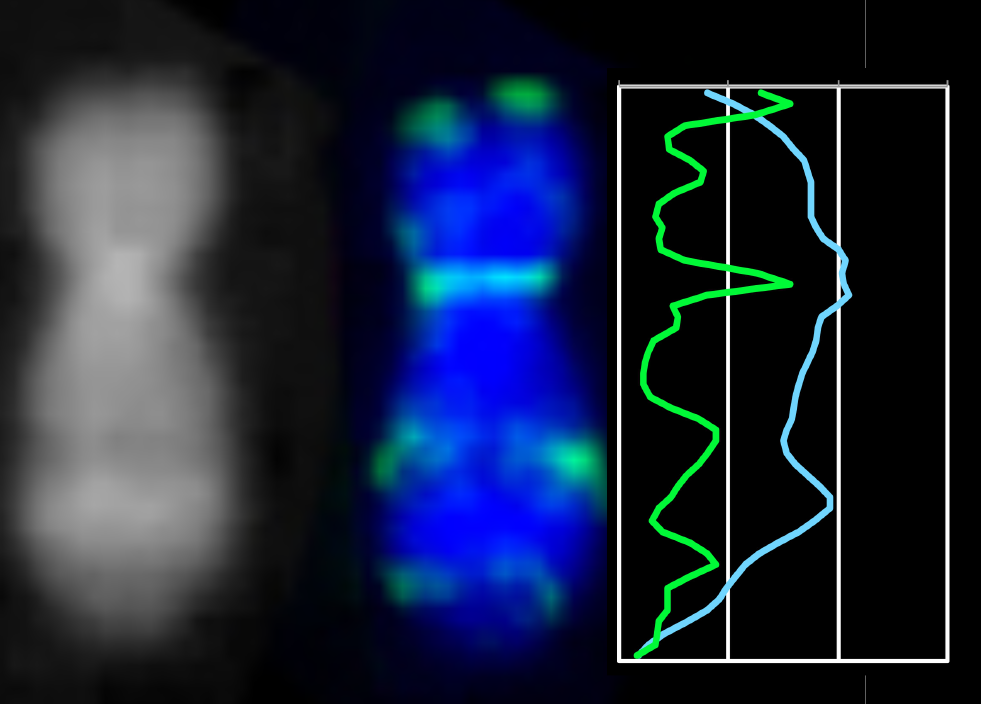

Supplement: Additional file 2: — Methylation pattern on chromosome 6 from Strain 4. The DAPI image is shown to the left of the immunostained image. Hypermethylation is observed either side of a darker staining DAPI band on the long arm, corresponding to added region. Line scan indicates intensity of methylation staining (green) and DAPI staining (blue). (TIFF 325 kb) [file 13039_2015_176_MOESM2_ESM.tif]

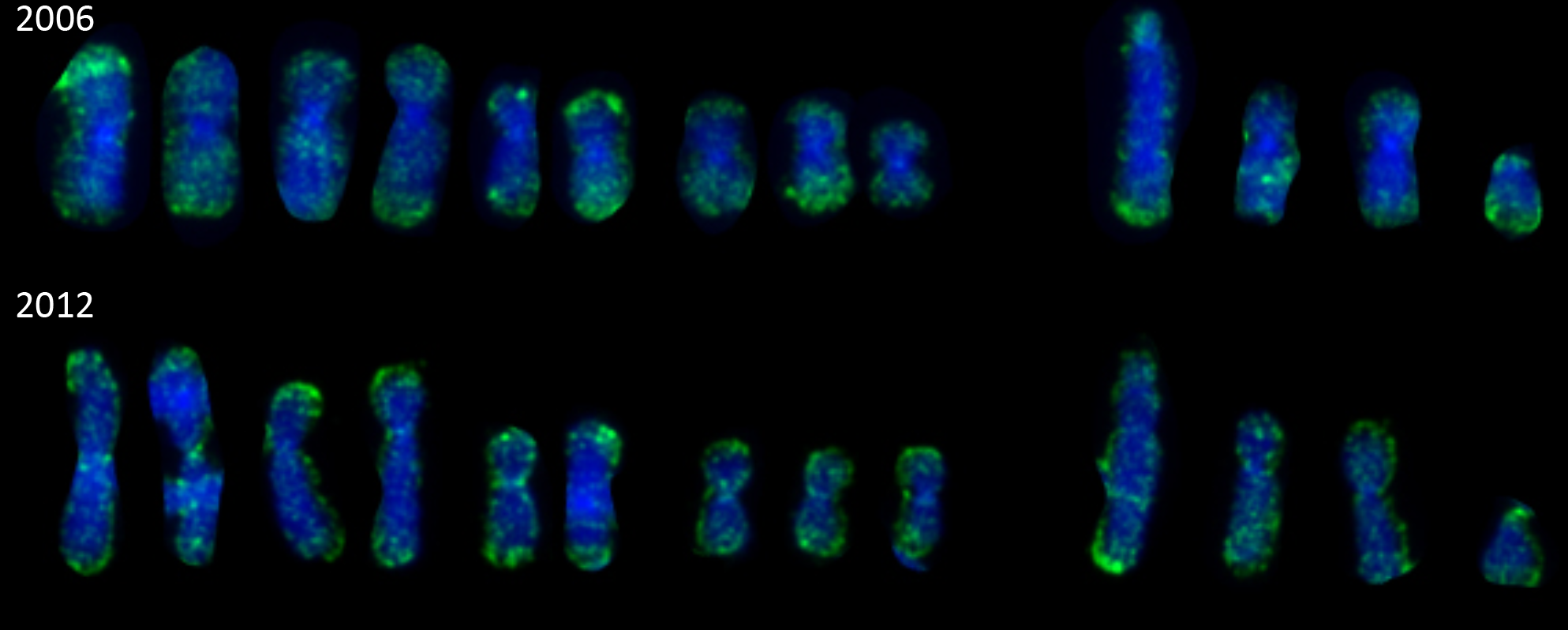

Supplement: Additional file 3: — Comparison of strain 1 global methylation between samples taken in 2006 and 2012. (TIFF 766 kb) [file 13039_2015_176_MOESM3_ESM.tif]
